# Effect of Combination Therapy with a Calcium Channel Blocker and an Angiotensin-Converting Enzyme Inhibitor on Renal Hypertrophy and Urinary Albumin Excretion in Diabetic Rats

**DOI:** 10.1155/EDR.2003.191

**Published:** 2003

**Authors:** Birgitte Nielsen, Henning Grønbæk, Ruth Østerby, Allan Flyvbjerg

**Affiliations:** 1 Medical Research Laboratories (Diabetes and Endocrinology) Institute of Experimental Clinical Research Aarhus University Hospital M-lab. II, Nørrebrogade 44 Aarhus DK-8000 Denmark; 2 Electron Microscopy Research Laboratory Institute of Experimental Clinical Research Aarhus University Hospital University Institute of Pathology Aarhus Denmark

## Abstract

The objective of this study was to compare the effect of an angiotensin-converting enzyme (ACE) inhibitor and a calcium channel blocker on the development of renal changes in diabetic rats. Diabetes was induced by an intravenous injection of streptozotocin in normotensive Wistar rats. Treatment was commenced immediately in 1 set of rats with 4 treatment arms: nitrendipine (250 mg/kg fodder), enalapril (35 mg/L drinking water), both treatments in combination, or placebo. Treatment was continued for 9 weeks. Another set of rats was left with untreated diabetes for 3 months followed by 7 weeks treatment as above. When starting treatment right after induction of diabetes, nitrendipine significantly reduced urinary albumin excretion (UAE) to the nondiabetic level (*P* < .05) without reducing blood pressure (BP), whereas enalapril failed to significantly reduce UAE despite a reduction in BP. Combining the two treatments showed no further reduction in UAE compared to monotherapy with nitrendipine, despite a lower BP. When leaving diabetic rats untreated for 3 months, only the coadministration of nitrendipine and enalapril showed a significant reduction in UAE compared to monotherapy and placebo treatment, but showed no significant effect on BP.


*Experimental Diab. Res.*, 4:191–199, 2003
Copyright © Taylor and Francis Inc.
ISSN: 1543-8600 print / 1543-8619 online
DOI: 10.1080/15438600390244362

### Effect of Combination Therapy with a Calcium Channel Blocker and an Angiotensin-Converting Enzyme Inhibitor on Renal Hypertrophy and Urinary Albumin Excretion in Diabetic Rats

**Birgitte Nielsen,[1] Henning Grønbæk,[1] Ruth Østerby,[2] and Allan Flyvbjerg[1]**

[1]*Medical Research Laboratories (Diabetes and Endocrinology), Institute of Experimental Clinical Research, Aarhus University Hospital, Aarhus, Denmark*
[2]*Electron Microscopy Research Laboratory, Institute of Experimental Clinical Research, Aarhus University Hospital, University Institute of Pathology, Aarhus, Denmark*

**The objective of this study was to compare the effect of an angiotensin-converting enzyme (ACE) inhibitor and a calcium channel blocker on the development of renal changes in diabetic rats. Diabetes was induced by an intravenous injection of streptozotocin in normotensive Wistar rats. Treatment was commenced immediately in 1 set of rats with 4 treatment arms: nitrendipine (250 mg/kg fodder), enalapril (35 mg/L drinking water), both treatments in combination, or placebo. Treatment was continued for 9 weeks. Another set of rats was left with untreated diabetes for 3 months followed by 7 weeks treatment as above. When starting treatment right after induction of diabetes, nitrendipine significantly reduced urinary albumin excretion (UAE) to the nondiabetic level ($P < .05$) without reducing blood pressure (BP), whereas enalapril failed to significantly reduce UAE despite a reduction in BP. Combining the two treatments showed no further reduction in UAE compared to monotherapy with nitrendipine, despite a lower BP. When leaving diabetic rats untreated for 3 months, only the coadministration of nitrendipine and enalapril showed a significant reduction in UAE compared to monotherapy and placebo treatment, but showed no significant effect on BP.**

**Keywords** Diabetes; Enalapril; Nitrendipiner; Rats; Renal Hypertrophy; Urinary Albumin Excretion

Diabetic nephropathy is the most common cause of end stage renal failure (ESRD) in the Western world [1], and is characterized by persistent proteinuria, hypertension, and declining renal function. Good metabolic control is essential in the prevention of diabetic nephropathy [2], but the treatment of hypertension with angiotensin-converting enzyme (ACE) inhibitors (ACEIs) has also proven effective in preventing or postponing the development of diabetic kidney disease [3–5]. The superior effect of ACEIs compared to conventional antihypertensive treatment with $\beta$-blockers and diuretics seems to be due to a renoprotective effect, i.e., a renal effect beyond the blood pressure (BP)-lowering effect [6–9]. However, other antihypertensive drugs, such as calcium channel blockers (CCBs), may also be effective in the treatment of diabetic kidney disease, although controversy still exists whether dihydropyridine CCBs and nondihydropyridine CCBs are equally effective in type 1 and type 2 diabetes mellitus [10–14]. In previous studies of

Received 2 May 2003; accepted 19 June 2003.
The authors thank L. Lysgaard, B. Saugbjerg, B. Iversen, K. Nyborg, K. Mathiassen, and N. Rosenquist for their technical assistance. The nitrendipine-containing fodder was a generous gift from Bayer, Germany. This study was supported by grants from the Aarhus University Research Foundation, The Novo Foundation, the Ingeborg Maibøll Foundation, the Danish Medical Research Council (no. 9700592), the Danish Kidney Foundation, the Ruth König Petersen Foundation, the Aage Louis-Hansen Memorial Foundation, the Nordic Insulin Foundation, The Eva and Henry Frænkels Memorial Foundation, and the Aarhus University–Novo Nordisk Center for Research in Growth and Regeneration (no. 9600822).
Address correspondence to Birgitte Nielsen, MD, PhD, Institute of Experimental Clinical Research, Aarhus University Hospital, Medical Research Laboratories, M-lab. II, Nørrebrogade 44, DK-8000 Aarhus C, Denmark. E-mail: BirgitteNielsen@dadlnet.dk experimentally diabetic rats, we showed that treatment with the dihydropyridine CCB nitrendipine was able to reduce urinary albumin excretion (UAE) and renal and glomerular hypertrophy when starting treatment early in the disease [15, 16]. In the present study, we compared the effect of the dihydropyridine CCB nitrendipine and the ACEI enalapril on renal changes in experimental diabetes in rats, focusing on UAE and renal and glomerular hypertrophy. The importance of the timing of intervention was investigated as well as a possible additive effect by combined treatment with CCB and ACEI. This study is the first to report the effect of combination therapy with a dihydropyridine CCB and an ACEI in a model of type 1 diabetes.

### MATERIAL AND METHODS

### Animals

One hundred and fifty-seven adult female Wistar rats (Møllegaards Avlslab, Eiby, Denmark), with an initial mean body weight of 200 ± 1 g, were randomized into 5 groups: nondiabetic placebo-treated control rats (CP), diabetic placebo-treated rats (DP), diabetic nitrendipine-treated rats (DN), diabetic enalapril-treated rats (DE), and diabetic nitrendipine- and enalapril-treated rats (DNE). All animal groups were assigned to 1 of 2 intervention trials: *prevention* with any of the antihypertensive treatment regimens starting at the time of diabetes induction and animals were killed after 9 weeks; *intervention*, leaving animals with untreated diabetes for 3 months followed by 7 weeks treatment. Diabetes was induced by a single intravenous (IV) injection of streptozotocin (STZ) (50 mg/kg body weight) (Upjohn Company, Kalamazoo, Michigan, USA) following 12 hours' overnight fasting. Animals were housed 2 to 3 animals per cage in a room with 12:12 hours' artificial light cycle, mean room temperature 21°C ± 2°C, and humidity 55% ± 2%. All principles of laboratory animal care and the current version of the Danish Law on Animal Experiments were followed. Nitrendipine (Bayer Company, Germany) was administered in the diet at a concentration of 250 mg/kg [15, 17], whereas enalapril (enalapril maleate; Sigma Chemicals, St. Louis, Missouri, USA) was given in drinking water at a concentration of 35 mg/L [6, 18]. Placebo-treated animal groups were given matched fodder with similar content of carbohydrates, protein, and fat, and regular tap water was administered. All animal groups had free access to rat chow and drinking water throughout the experiment. Nitrendipine-containing fodder was covered by metal plates to avoid light exposure, and fodder was changed every 2nd or 3rd day. Enalapril-containing water was changed every other day. Throughout the experiment, animals were weighed, food consumption was measured, and tail-vein blood glucose levels were determined by Haemoglucotest 1–44 and Reflolux II reflectance meter (Boehringer-Mannheim, Mannheim, Germany). Urinalysis was performed by simple dip-sticks for glucose and ketones (Neostix-4, Ames, Stoke Poges, Slough, UK). Three times during the study, all animals were placed in metabolic cages for 24-hour urine collection for determination of UAE (see below). Six control animals in each intervention trial were taken out for investigation at the beginning of the study as a reference group (CP0). Following anesthesia with mebumal (50 mg/kg body weight, intraperitoneal [IP]), blood was drawn from the retro-orbital venous plexus. Both kidneys were taken out and the capsules gently removed. Kidneys were weighed, and the left kidney was fixed in 3% formaldehyde and 1% glutaraldehyde in modified Tyrode buffer overnight [15, 16] for later morphometric analysis (see below).

In the prevention trial (animal groups with suffix "P"), 81 animals were included at study start, and 69 animals fulfilled the study, n: $CP_P = 21$ (6 animals investigated at study start, $CP0_P$), $DP_P = 10$, $DN_P = 12$, $DE_P = 14$, $DNE_P = 12$. Similarly, in the intervention trial (animal groups with suffix "I"), 76 animals were included with 44 animals fulfilling the study, n: $CP_I = 14$ (6 animals investigated at study start, $CP0_I$), $DP_I = 9$, $DN_I = 7$, $DE_I = 8$, $DNE_I = 6$. The exclusion criteria were persistent ketonuria and weight loss, blood glucose levels below 18 mmol/L, glucosuria below 111 mmol/L, death, or the finding of pyelonephritis at study termination. Only data from animals fulfilling these study criteria were included.

### Blood Pressure Measurements

By the end of each treatment trial, systolic BP was measured in 6 to 7 randomly selected animals in each group by the tail cuff method as previously described [19]. All measurements were performed in the early afternoon to avoid 24-hour BP fluctuations. Animals were placed in an acrylic container, and after approximately 10 minutes' acclimatization, 5 consecutive measurements were performed, and a mean value was determined.

### Urinary Albumin Excretion

Twenty-four-hour UAE was determined by radioimmunoassay as previously described [20], using rat antibody and standards. Urine samples were stored at −20°C until analysis. Rabbit anti-rat albumin antibody (RAR/Alb) was purchased from Nordic Pharmaceuticals and Diagnostics (Tilburg, The Netherlands). For standard and iodination, a globulin-free rat albumin was obtained from Sigma Chemicals.

### Serum Fructosamine

Reagents and standards for the fructosamine assay, Fructosamine Test Plus, were purchased from Hoffmann-La Roche (Basel, Switzerland) and the determination was performed as previously described [21]. Serum samples were stored at −20°C until assayed.

### Kidney Morphology

After overnight fixation, the left kidney was cut into slices of 2-mm thickness by a set of fixed razor blades. The slices were embedded in paraffin and sections were cut at 2 levels with a distance of 250 $\mu$m and stained with the periodic acid–Schiff reaction. This procedure provides a set of independently positioned sections, which were used for measurements by standard stereological methods [15, 16, 22] using point counting for determination of glomerular volume fraction. A grid with coarse points:fine points 1:4 was used. All sections were counted blinded by the same observer. Total glomerular volume was estimated as the product of volume fraction and kidney weight, assuming that 1 mg kidney tissue equals 1 mm$^3$.

### Statistical Analyses

Differences between normally distributed data were compared among groups by analysis of variance (ANOVA), and when a significant effect was identified, an unpaired $t$ test was performed. The values of UAE were logarithmically transformed due to their positively skewed distribution. A $P$ value ≤.05 in a 2-tailed test was considered indicative of a statistically significant difference. Only data within the same trial were compared. All values are given as mean ± standard error of the mean (SEM). When multiple comparisons were performed, the Bonferroni correction was used.

### RESULTS

### Body Weight and Food Consumption

Body weights at study termination are shown in Table 1. Although body weight was steadily increasing in nondiabetic control animals, a marked growth retardation was observed in all diabetic animal groups with lesser increase compared to nondiabetic animals ($P < .05$). This growth retardation was observed in both the prevention (Table 1A) and the intervention (Table 1B) trials. However, no significant difference was seen between any of the diabetic groups in the two treatment arms, indicating that neither nitrendipine nor enalapril showed

**TABLE 1**
Body weight (g), serum fructosamine ($\mu$mol/L), and systolic blood pressure (mm Hg) in prevention and intervention trials

A. Prevention trial (9 weeks)

| Animal group | Body weight Day 0 | Body weight 9 weeks | Serum fructosamine Day 0 | Serum fructosamine 9 weeks | Blood pressure 9 weeks |
|---|---|---|---|---|---|
| $CP_P$ (n = 15) | 199 ± 2 | 250 ± 5* | 218 ± 5 | 216 ± 6# | 110 ± 1 |
| $DP_P$ (n = 10) | 199 ± 6 | 229 ± 7 | | 274 ± 11 | 112 ± 1 |
| $DN_P$ (n = 12) | 197 ± 4 | 219 ± 4 | | 280 ± 7 | 112 ± 1 |
| $DE_P$ (n = 14) | 203 ± 8 | 214 ± 5 | | 292 ± 10 | 97 ± 1† |
| $DNE_P$ (n = 12) | 201 ± 4 | 222 ± 4 | | 292 ± 5 | 97 ± 1† |

B. Intervention trial (3 months and 7 weeks)

| Animal group | Body weight Day 0 | Body weight 3 mo + 7 wk | Serum fructosamine Day 0 | Serum fructosamine 3 mo + 7 wk | Blood pressure 3 mo + 7 wk |
|---|---|---|---|---|---|
| $CP_I$ (n = 8) | 211 ± 2 | 255 ± 4** | 224 ± 4 | 221 ± 6## | 97 ± 4 |
| $DP_I$ (n = 9) | 200 ± 2 | 223 ± 4 | | 308 ± 10 | 106 ± 4 |
| $DN_I$ (n = 7) | 195 ± 2 | 226 ± 10 | | 303 ± 10 | 112 ± 3 |
| $DE_I$ (n = 8) | 204 ± 4 | 232 ± 5 | | 287 ± 7 | 94 ± 3 |
| $DNE_I$ (n = 6) | 201 ± 3 | 235 ± 7 | | 319 ± 11 | 102 ± 2 |

$CP_P/CP_I$: nondiabetic control placebo-treated rats; $DP_P/DP_I$: diabetic placebo-treated rats; $DN_P/DN_I$: diabetic nitrendipine-treated rats; $DE_P/DE_I$: diabetic enalapril-treated rats; $DNE_P/DNE_I$: diabetic nitrendipine/enalapril-treated rats. Values are expressed as mean ± SEM.

*$P < .05$, $CP_P$ versus diabetic animals in the prevention trial.

#$P < .001$, $CP_P$ versus diabetic animals in the prevention trial.

†$P < .05$, $DE_P$ and $DNE_P$ versus $DP_P$.

**$P < .05$, $CP_I$ versus diabetic animals in the intervention trial.

##$P < .01$, $CP_I$ versus diabetic animals in the intervention trial.

any impact on body weight. Also, the number of animals excluded during the study was comparable between treated and nontreated diabetic groups, although more animals assigned to the intervention trial were excluded, probably due to severe hyperglycemia for a prolonged period of time. Diabetic animals revealed hyperphagia 1 week after diabetes induction when compared to nondiabetic animals, but no significant difference in food consumption was observed between treated or nontreated diabetic animals. Daily food intake in all diabetic animals averaged 28 ± 2 g/24 hours throughout the experiments, whereas food intake in nondiabetic animals averaged 16 ± 2 g/24 hours (data not shown). Thus, because all rat chows were matched, no significant difference in protein intake was observed between diabetic rats. Finally, all diabetic rats developed polydipsia, but the exact water consumption was not measured in these studies.

### Metabolic Parameters

*Blood Glucose*

Within 24 hours after STZ injections, diabetic animals were hyperglycemic, with blood glucose levels averaging 25 ± 0.5 mmol/L. All groups stabilized at this level throughout the study, and antihypertensive treatment showed no influence on blood glucose. Blood glucose in nondiabetic animals averaged 4.6 ± 0.3 mmol/L (data not shown).

*Serum Fructosamine*

Fructosamine was assayed when terminating the trials, and is shown in Table 1. All diabetic groups showed significantly increased serum fructosamine compared to nondiabetic animals ($P < .001$), but no difference was detected among diabetic groups, showing that neither treatment had any influence on serum fructosamine.

### Blood Pressure

Systolic BP measured by the end of the observation periods is shown in Table 1. In the prevention trial, the diabetic state did not lead to increased systemic BP in placebo treated animals after 9 weeks (Table 1A). However, enalapril-treatment significantly reduced BP compared to placebo treatment ($P < .001$), whereas no further reduction in BP was detected when combining enalapril and nitrendipine. When nitrendipine was administered as monotherapy, no significant reduction in BP was observed compared to placebo-treated diabetic animals. Thus, only enalapril led to a reduction in systemic BP, although all animals were normotensive when the study was terminated.

In the intervention trial, no significant difference in BP was observed between nondiabetic and any of the diabetic groups (Table 1B). Although enalapril treatment as monotherapy and in combination with nitrendipine reduced systemic BP in the prevention trial, this reduction was not observed in the intervention trial.

### Kidney Weight

Figure 1 shows kidney weight in non-diabetic animals at study start (CP0), and in all other groups at study termination in the prevention (Figure 1*A*) and the intervention trials (Figure 1*B*). Diabetes induction was followed by highly significant renal hypertrophy compared to the nondiabetic condition ($P < .001$). No significant difference in kidney weight was observed between placebo-treated diabetic animals and diabetic animals assigned to any of the antihypertensive treatment regimens ($P > .05$) in neither prevention nor intervention trials.

### Glomerular Volume

Total glomerular volumes (TGV) are illustrated in Figure 2. In both the prevention (Figure 2*A*) and the intervention (Figure 2*B*) trials, diabetes was associated with a significant increase in TGV compared to the nondiabetic condition ($P < .05$), and neither monotherapy nor combined treatment with nitrendipine and enalapril showed any impact on diabetes-induced glomerular hypertrophy compared to placebo treatment ($P > .05$).

### Urinary Albumin Excretion

In the prevention trial, UAE was measured 3 times during the study period and is presented in Figure 3*A*. One month following diabetes induction, all diabetic animals showed a significant increase in UAE compared to nondiabetic animals ($P < .05$). Although UAE was lower in treated animal groups after 1 month compared to placebo-treated diabetic animals, this did not reach statistical significance (Figure 3*A*). After 9 weeks, UAE was reduced to the nondiabetic level in nitrendipine-treated rats, and was significantly different from placebo-treated diabetic animals ($P < .05$). Combining enalapril and nitrendipine treatment showed no further reduction in UAE compared to monotherapy with nitrendipine, and monotherapy with enalapril failed to reveal a significant reduction in UAE compared to placebo-treated diabetic animals, although a tendency toward a lower UAE was observed ($P > .05$).

In the intervention trial, a highly significant increase in UAE was observed after 3 months of untreated diabetes compared to the nondiabetic condition ($P < .001$) (Figure 3*B*). Following 7 weeks of treatment with nitrendipine and/or enalapril, only nitrendipine and enalapril given in combination significantly reduced UAE compared to untreated diabetic animals ($P < .05$), whereas monotherapy with either regimen for 7 weeks

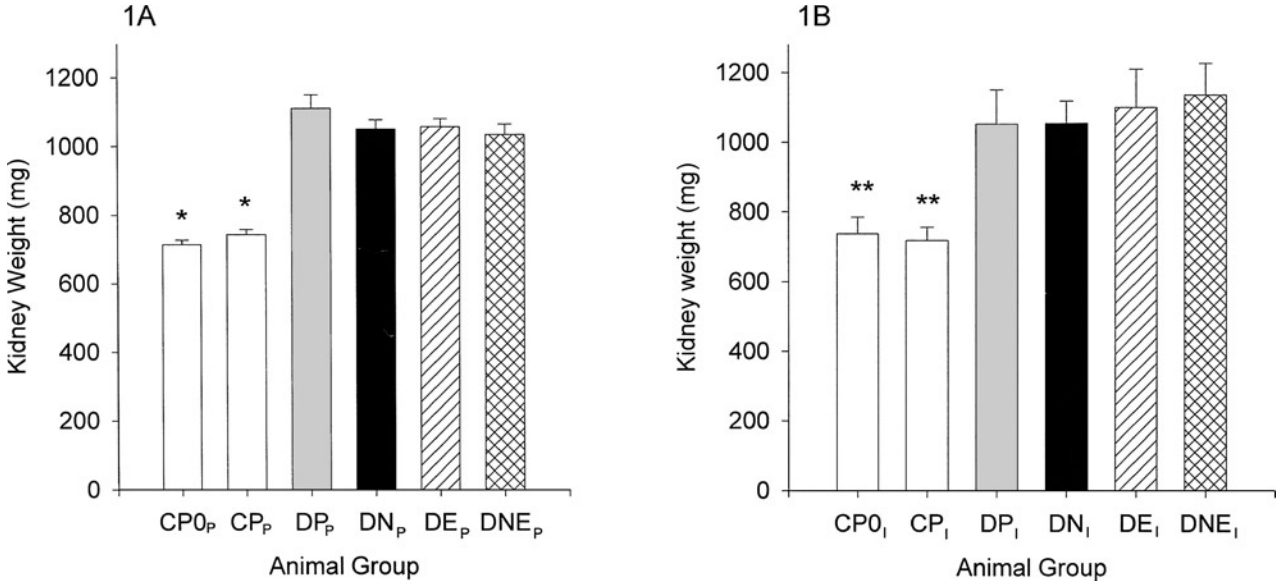

**FIGURE 1**

Right kidney weights in all animal groups are illustrated in mg. Prevention trial (*A*) and intervention trial (*B*). $CP0_P/CP0_I$: nondiabetic control placebo-treated rats at study start; $CP_P/CP_I$: nondiabetic control placebo-treated rats at end of study; $DP_P/DP_I$: diabetic placebo-treated rats; $DN_P/DN_I$: diabetic nitrendipine-treated; $DE_P/DE_I$: diabetic enalapril-treated; $DNE_P/DNE_I$: diabetic nitrendipine/enalapril-treated. Values are shown as mean ± SEM. $^{*}P < .001$, $CP_P$ versus diabetic animal groups in the prevention trial. $^{**}P < .001$, $CP_I$ versus diabetic animal groups in the intervention trial.

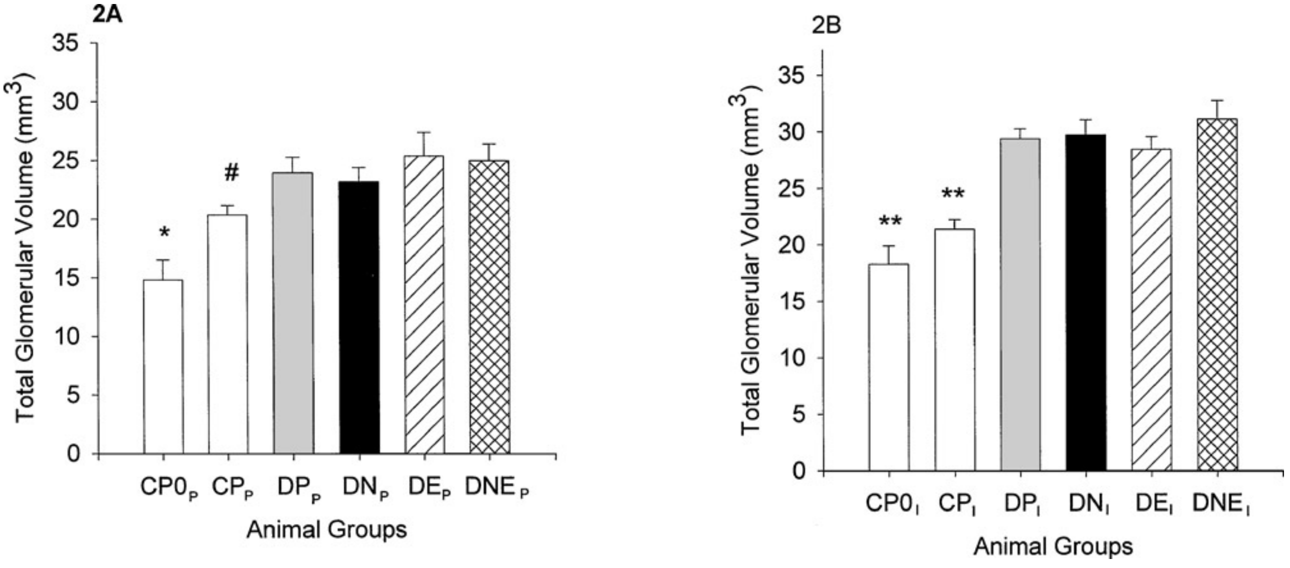

**FIGURE 2**

Total glomerular volume in the prevention trial (*A*) and the intervention trial (*B*). $CP0_P/CP0_I$: nondiabetic control placebo-treated rats at study start; $CP_P/CP_I$: nondiabetic control placebo-treated rats at end of study; $DP_P/DP_I$: diabetic placebo-treated rats; $DN_P/DN_I$: diabetic nitrendipine-treated; $DE_P/DE_I$: diabetic enalapril-treated; $DNE_P/DNE_I$: diabetic nitrendipine/enalapril-treated. Values are shown as mean ± SEM. $^{*}P < .05$, $CP0_P$ versus $CP_P$; $^{\#}P < .05$, $CP_P$ versus $DP_P$; $^{**}P < .05$, $CP0_I$ and $CP_I$ versus $DP_I$.

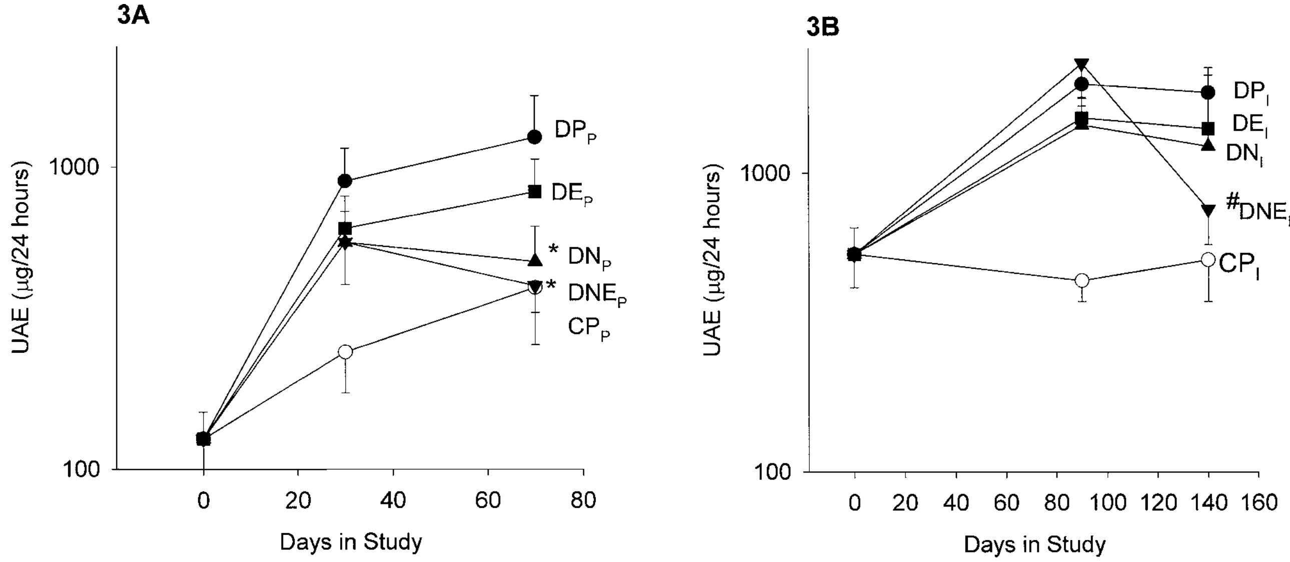

**FIGURE 3**

Urinary albumin excretion shown on a logarithmic scale. Prevention trial (*A*) and intervention trial (*B*). $CP_P/CP_I$: nondiabetic control placebo-treated rats at end of study; $DP_P/DP_I$: diabetic placebo-treated rats; $DN_P/DN_I$: diabetic nitrendipine-treated; $DE_P/DE_I$: diabetic enalapril-treated; $DNE_P/DNE_I$: diabetic nitrendipine/enalapril-treated. Values are shown as mean ± SEM. $^*P < .05$, $DN_P$ and $DNE_P$ versus $DP_P$; $^\#P < .05$, $DNE_I$ versus $DP_I$.

failed to reduce UAE significantly after 3 months of untreated diabetes (Figure 3*B*).

### DISCUSSION

In the present study, we investigated whether CCB treatment was as effective as ACEI treatment in preventing the development of diabetic kidney disease in STZ diabetic Wistar rats. The importance of time of intervention was investigated by comparing prevention and intervention. Also, a potential additive effect of combining the 2 treatment arms was investigated.

We found that monotherapy with the dihydropyridine CCB nitrendipine reduced the diabetes-associated increase in UAE when starting treatment right at the time of diabetes induction (prevention), whereas no such effect was observed with the ACEI enalapril, despite that a lower BP was attained with ACEI treatment. Postponing treatment until diabetic renal changes were manifest after 3 months of untreated diabetes (intervention), only the combination of CCB and ACEI was able to reduce UAE, whereas no such effect was observed by either treatment alone. These results were observed without any influence on metabolic control and renal or glomerular hypertrophy. The results obtained with nitrendipine treatment alone are in accordance with our previous studies [15, 16, 22] reporting nitrendipine to reduce UAE in diabetic rats, when starting treatment early in the disease, without a significant effect on kidney size and glomerular hypertrophy [16, 22]. In the present study, BP was only significantly lowered in animal groups treated with ACEI or ACEI/CCB in the prevention trial. In the intervention trial, BP was also lower in ACEI-treated animals, but due to much greater variability in BP within the individual groups after 3 months and 7 weeks, this did not reach statistical significance. We used the tail-cuff method for detecting systolic BP, because the animals in this study were severely hyperglycaemic, and thus sensitive to invasive procedures. However, previous studies have shown that antihypertensive treatment may abolish the normal circadian rhythm of BP [23], which limits the assessment of ambient systemic BP using the tail-cuff method, although the diurnal variation was reduced by performing all BP measurements during early afternoon. The BP-lowering effect of ACE inhibition and no effect on BP due to calcium channel blockade have been reported previously in experimental studies in normotensive diabetic Wistar rats [6, 15, 16, 18, 22, 24].

Several clinical [3, 4, 25–27] and experimental [28–30] studies have shown that ACEI treatment is able to prevent or postpone the development of diabetic kidney disease, and ACEIs have become the drugs of first choice when initiating antihypertensive treatment in microalbuminuric diabetic patients [31]. However, our present study failed to demonstrate any effect of enalapril on diabetes-associated renal changes, i.e., renal and glomerular hypertrophy and albuminuria, which is not explained by insufficient drug dosage, because a significant reduction in systolic BP was demonstrated. Also, in the intervention trial of this study, only the combination of CCB and ACEI treatment showed inhibitory effects on renal changes compared to monotherapy with either drug, further indicating that enalapril was given in a adequate dosage, and in accordance with previous studies [6, 18]. Thus, biologic variation and the relatively large day-to-day variation in UAE, which was particular high in the group in which enalapril was given as monotherapy, may explain why no significant effect was observed on UAE in this group. Despite having 14 animals fulfill the study criteria in the enalapril-treated group in the prevention trial, the great variability in UAE may have caused a statistical type II error. The determination of UAE was performed by a well-described radioimmunoassay [20] and in accordance with previous studies [6, 14, 15, 22, 24], thus the lack of an effect on albuminuria in the enalapril-treated group is not believed to be due to methodological errors. However, it would have been warranted to collect 24-hour urine in a consecutive number of days to minimize the variability. Despite the lack of an antialbuminuric effect observed by enalapril treatment and the discrepancy with other reports [6, 18], the results of the present study are important, because CCB treatment was shown to be at least as effective as ACEI treatment in slowing the progression of experimental diabetic kidney disease, using UAE as an end-point parameter. More importantly, it was demonstrated in the intervention trial that in a more advanced state of diabetic kidney disease, only the combination of ACE-inhibition and calcium channel blockade achieved a reduction in UAE. Thus, despite the variability in BP in the prevention and intervention trials, and the lack of an antiproteinuric effect following enalapril treatment, the hypothesis that ACEI and CCB treatments show additive effects in the prevention of diabetic kidney disease was demonstrated. Although UAE was lowered by nitrendipine in the prevention trial, and by combination therapy in the intervention trial, no effect on renal and glomerular hypertrophy was observed at any time. Previous studies in experimental diabetes also report lowering effects on UAE with unchanged renal enlargement following CCB therapy [16] and ACEI treatment [30].

Several studies have investigated the effect of both dihydropyridine and nondihydropyridine CCBs on the progression of diabetic kidney disease. However, the studies are very heterogeneous, investigating type 1 or type 2 diabetic patients with either incipient or overt diabetic nephropathy, or in the case of investigating STZ diabetic animals, using rats of different species, or diabetic Beagle dogs; for review see [14]. Also, results are divergent and conflicting results and much attention has been paid to the use and safety of CCBs in both type 1 and type 2 diabetic patients after the premature termination of the ABCD trial [32] and the FACET study [33]. The superior effect of ACEIs compared to other antihypertensive drugs has been ascribed a renoprotective effect, i.e., an effect that is beyond the effect caused by a reduction in systemic BP. Studies have reported an inhibition of diabetic renal changes irrespective of systemic BP reduction, indicating a direct effect on intrarenal hemodynamics [3–5, 27]. ACEIs have a dilating effect on the glomerular efferent arteriole, and thus reduce the intraglomerular hydraulic pressure [34, 35], which may explain the renoprotective effect observed without a concomitant reduction in systemic BP. In contrast to ACEIs, CCBs intrarenal effect is believed to be a dilating effect on the afferent more than on the efferent arteriole [36, 37], and theoretically this should induce an increase in glomerular capillary pressure. A renoprotective effect is thus dependent on a concomitant reduction in systemic BP. However, both ACEIs and CCB may possess nonvascular effects. In vitro studies have shown that the mitogenic effect of several growth factors are attenuated by both ACEIs [6, 30, 38] and CCBs [39], and both groups of drugs have been reported to preserve glomerular heparan sulfate [9, 40]. Thus, despite a greater dilating effect on the afferent arteriole than on the efferent arteriole attained by CCB treatment, the nonvascular effects could be very important. In recent papers, it has been suggested that BP reduction to a level of 125–130/80–85 mm Hg may be more important than the actual drug chosen in the treatment of diabetic patients [41–44]. Reducing BP to this level in hypertensive patients, diabetic as well as nondiabetic subjects, may only be attainable with the combination of 2 or more antihypertensive drugs. Because ACEIs reduce the generation of angiotensin II (AT-II), and CCBs reduce target-organ responsiveness to AT-II [37, 45], these two groups of antihypertensive drugs may be preferable to conventional therapy in diabetic patients, either as monotherapy, or administered in combination.

In the present study, we demonstrated a BP reduction following monotherapy with enalapril, whereas no effect on UAE was observed, thus, confirming that diabetic kidney disease is caused by other factors besides systemic BP. This was consolidated by the fact that nitrendipine lowered UAE compared to enalapril treatment, despite that nitrendipine-treated animals showed a significantly higher BP than enalapril-treated animals. Also, no difference in metabolic parameters between the different trials was observed. However, potential nonvascular mechanisms were not investigated in this study.

It has been claimed in several papers by Bakris and colleagues that only nondihydropyridine CCBs show a protection against diabetic renal disease [10, 12, 46–48]. However, only 1 long-term clinical study from another group has investigated the effect of a dihydropyridine CCB, namely nisoldipine, and found CCB treatment to preserve glomerular filtration rate (GFR) to the same extent as ACEI (lisinopril) treatment, despite a better antiproteinuric effect observed with lisinopril over a 4-year study period [11]. As stated in this article [11], GFR is a better end-point parameter than UAE when evaluating renal function.

Thus, because the goal of BP reduction in diabetic patients may not be attainable with monotherapy with ACEI, the results of the present study and the study mentioned above [11] indicate that the combination of dihydropyridine CCB and ACEI treatment may be a potential treatment of diabetic patients with hypertension with or without nephropathy.

In conclusion, the dihydropyridine CCB nitrendipine attenuates the development of albuminuria in normotensive diabetic rats without a reduction in systemic BP when starting treatment at diabetes debut. If postponing treatment until renal changes are present, only the combination of a CCB and an ACEI shows a reduction in UAE compared to either treatment alone. The impact of combination therapy on both hemodynamic and nonhemodynamic parameters needs further investigation in clinical as well as experimental studies.

### REFERENCES

[1] Bilous, R. W., and Marshall S. M. (1997) Clinical aspects of nephropathy. In: *International Textbook of Diabetes Mellitus,* Edited by Alberti, K. G. M. M., Zimmet, P., and DeFronzo, R. A., pp. 1363–1411. Chichester, UK: John Wiley & Sons.

[2] The Diabetes Control and Complications Trial Research Group. (1993) The effect of intensive treatment of diabetes on the development and progression of long-term complications in insulin-dependent diabetes mellitus. *N. Engl. J. Med.,* **329,** 977–986.

[3] Lewis, E. J., Hunsicker, L. G., Bain, R. P., and Rohde, R. D. (1993) The effect of angiotensin-converting enzyme inhibition on diabetic nephropathy. The Collaborative Study Group. *N. Engl. J. Med.,* **329,** 1456–1462.

[4] Björck, S., Mulec, H., Johnsen, S. A., Norden, G., and Aurell, M. (1992) Renal protective effect of enalapril in diabetic nephropathy. *BMJ,* **304,** 339–343.

[5] Marre, M., Chatellier, G., Leblanc, H., Guyene, T. T., Menard, J., and Passa, P. (1988) Prevention of diabetic nephropathy with enalapril in normotensive diabetics with microalbuminuria. *BMJ,* **297,** 1092–1095.

[6] Hill, C., Logan, A., Smith, C., Grønbæk, H., and Flyvbjerg, A. (2001) Angiotensin converting enzyme inhibitor suppresses glomerular transforming growth factor $\beta$ receptor expression in experimental diabetes in rats. *Diabetologia,* **44,** 495–500.

[7] Ray, P. E., Aguilera, G., Kopp, J. B., Horikoshi, S., and Klotman, P. E. (1991) Angiotensin II receptor-mediated proliferation of cultured human fetal mesangial cells. *Kidney Int.,* **40,** 764–771.

[8] Wolf, G., Haberstroh, U., and Neilson, E. G. (1992) Angiotensin II stimulates the proliferation and biosynthesis of type I collagen in cultured murine mesangial cells. *Am. J. Pathol.,* **140,** 95–107.

[9] Reddi, A. S., Ramamurthi, R., Miller, M., Dhuper, S., and Lasker, N. (1991) Enalapril improves albuminuria by preventing glomerular loss of heparan sulfate in diabetic rats. *Biochem. Med. Metab. Biol.,* **45,** 119–131.

[10] Bakris, G. L., Weir, M. R., DeQuattro, V., and McMahon, G. (1998) Effects of an ACE inhibitor/calcium antagonist combination on proteinuria in diabetic nephropathy. *Kidney Int.,* **54,** 1283–1289.

[11] Tarnow, L., Rossing, P., Jensen, C., Hansen, B. V., and Parving, H.-H. (2000) Long-term renoprotective effect of nisoldipine and lisinopril in type I diabetic patients with diabetic nephropathy. *Diabetes Care,* **23,** 1725–1730.

[12] Abbott, K., Smith, A., and Bakris, G. L. (1996) Effects of dihydropyridine calcium antagonists on albuminuria in patients with diabetes. *J. Clin. Pharmacol.,* **36,** 274–279.

[13] Jungmann, E., Malanyn, M., Mortasawi, N., Unterstoger, E., Haak, T., Palitzsch, K. D., Scherberich, J., Schumm Draeger, P. M., and Usadel, K. H. (1994) Effect of 1-year treatment with nitrendipine versus enalapril on urinary albumin and alpha 1-microglobulin excretion in microalbuminuric patients with type 1 diabetes mellitus. A randomized, single-blind comparative study. *Arzneimittelforschung,* **44,** 313–317.

[14] Nielsen, B., and Flyvbjerg, A. (2000) Calcium channel blockers—the effect on renal changes in clinical and experimental diabetes: An overview. *Nephrol. Dial. Transplant.,* **15,** 581–585.

[15] Nielsen, B., Grønbæk, H., Østerby, R., Ørskov, H., and Flyvbjerg, A. (1999) The calcium channel blocker nitrendipine attenuates renal and glomerular hypertrophy in diabetic rats. *Exp. Nephrol.,* **7,** 242–250.

[16] Nielsen, B., Grønbæk, H., Østerby, R., and Flyvbjerg, A. (2000) Effect of nitrendipine and nisoldipine on renal structure and function in long-term experimental diabetes in rats. *Am. J. Kidney Dis.,* **36,** 368–377.

[17] Fogo, A., and Ichikawa, I. (1989) Evidence for the central role of glomerular growth promoters in the development of sclerosis. *Semin. Nephrol.,* **9,** 329–342.

[18] Cooper, M. E., Allen, T. J., Macmillan, P. A., Clarke, B. E., Jerums, G., and Doyle, A. E. (1989) Enalapril retards glomerular basement membrane thickening and albuminuria in the diabetic rat. *Diabetologia,* **32,** 326–328.

[19] Pfeffer, J. M., Pfeffer, M. A., and Frohlich, E. D. (1971) Validity of an indirect tail-cuff method for determining systolic arterial pressure in unanesthetized normotensive and spontaneously hypertensive rats. *J. Lab. Clin. Med.,* **78,** 957–962.

[20] Christensen, C., and Ørskov, C. (1984) Rapid screening PEG radioimmunoassay for quantification of pathological microalbuminuria. *Diabetic Nephrol.,* **3,** 92–94.

[21] Boye, N., and Ingerslev, J. (1988) Rapid and inexpensive microdetermination of serum fructosamine results in diabetics, uraemics, diabetics with uraemia and healthy subjects. *Scand. J. Clin. Lab. Invest.,* **48,** 779–783.

[22] Nielsen, B., Grønbæk, H., Østerby, R., and Flyvbjerg, A. (1999) Effect of the calcium channel blocker nitrendipine in normotensive and spontaneously hypertensive, diabetic rats on kidney morphology and urinary albumin excretion. *J. Hypertens.,* **17,** 973–981.

[23] Griffin, K. A., Picken, M., and Bidani, A. K. (1994) Radiotelemetric BP monitoring, antihypertensives and glomeruloprotection in remnant kidney model. *Kidney Int.,* **46,** 1010–1018.

[24] Grønbæk, H., Vogel, I., Østerby, R., Lancranjan, I., Flyvbjerg, A., and Ørskov, H. (1998) Effect of octreotide, captopril or insulin on renal changes and UAE in long-term experimental diabetes. *Kidney Int.,* **53,** 173–180.

[25] Pedersen, M. M., Hansen, K. W., Schmitz, A., Sørensen, K., Christensen, C. K., and Mogensen, C. E. (1992) Effects of ACE

inhibition supplementary to beta blockers and diuretics in early diabetic nephropathy. *Kidney Int.,* **41,** 883–890.

[26] Elving, L. D., Wetzels, J. F., van Lier, H. J., de Nobel, E., and Berden, J. H. (1994) Captopril and atenolol are equally effective in retarding progression of diabetic nephropathy. Results of a 2-year prospective, randomized study. *Diabetologia,* **37,** 604–609.

[27] Rudberg, S., Aperia, A., Freyschuss, U., and Persson, B. (1990) Enalapril reduces microalbuminuria in young normotensive type 1 (insulin-dependent) diabetic patients irrespective of its hypotensive effect. *Diabetologia,* **33,** 470–476.

[28] Perico, N., Amuchastegui, S. C., Colosio, V., Sonzogni, G., Bertani, T., and Remuzzi, G. (1994) Evidence that an angiotensin-converting enzyme inhibitor has a different effect on glomerular injury according to the different phase of the disease at which the treatment is started. *J. Am. Soc. Nephrol.,* **5,** 1139–1146.

[29] Hajinazarian, M., Cosio, F. G., Nahman, N. S., and Mahan, J. D. (1994) Angiotensin-converting enzyme inhibition partially prevents diabetic organomegaly. *Am. J. Kidney Dis.,* **23,** 105–117.

[30] Gilbert, R. E., Cox, A., Wu, L. L., Allen, T. J., Hulthen, L., Jerums, G., and Cooper, M.E. (1998) Expression of transforming growth factor-$\beta$1 and type IV collagen in the renal tubulointerstitium in experimental diabetes. *Diabetes,* **47,** 414–422.

[31] Mogensen, C. E., Keane, W. F., Bennett, P. H., Jerums, G., Parving, H. H., Passa, P., Steffes, M. W., Striker, G. E., and Viberti, G. C. (1995) Prevention of diabetic renal disease with special reference to microalbuminuria. *Lancet,* **346,** 1080–1084.

[32] Estacio, R. O., Jeffers, B. W., Hiatt, W. R., Biggerstaff, S., Giffort, N., and Schrier, R. W. (1998) The effect of nisoldipine as compared with enalapril on cardiovascular outcomes in patients with non-insulin-dependent diabetes and hypertension. *N. Engl. J. Med.,* **338,** 645–652.

[33] Tatti, P., Pahor, M., Byington, R. P., Di Mauro, P., Guarisco, R., Strollo, G., and Strollo, F. (1998) Outcome results of the fosinopril versus amlodipine cardiovascular events randomized trial (FACET) in patients with hypertension and NIDDM. *Diabetes Care,* **21,** 597–603.

[34] Anderson, S., Rennke, H. G., and Brenner, B. M. (1992) Nifedipine versus fosinopril in uninephrectomized diabetic rats. *Kidney Int.,* **41,** 891–897.

[35] Zatz, R., Dunn, B. R., Meyer, T. W., Anderson, S., Rennke, H. G., and Brenner, B. M. (1986) Prevention of diabetic glomerulopathy by pharmacological amelioration of glomerular capillary hypertension. *J. Clin. Invest.,* **77,** 1925–1930.

[36] Ruggenenti, P., Mosconi, L., Bianchi, L., Cortesi, L., Campana, M., Pagani, G., Mecca, G., and Remuzzi, G. (1994) Long-term treatment with either enalapril or nitrendipine stabilizes albuminuria and increases glomerular filtration rate in non-insulin-dependent diabetic patients. *Am. J. Kidney Dis.,* **24,** 753–761.

[37] Carmines, P. K., and Navar, L. G. (1989) Disparate effects of Ca channel blockade on afferent and efferent arteriolar responses to ANG II. *Am. J. Physiol.,* **256,** F1015–F1020.

[38] Raij, L. (1995) Effects of ACE inhibitors and calcium channel blockers on the development of glomerular sclerosis in models of renal damage. *Nephrol. Dial. Transplant.,* **10**(Suppl 9), 23–27.

[39] Shultz, P., and Raij, L. (1992) Effect of amlodipine on mesangial cell proliferation and protein synthesis. *Am. J. Hypertens.,* **5,** 912–914.

[40] Jyothirmayi, G. N., and Reddi, A. S. (1993) Effect of diltiazem on glomerular heparan sulfate and albuminuria in diabetic rats. *Hypertension,* **21,** 795–802.

[41] Hansson, L., Zanchetti, A., Carruthers, S. G., Dahlöf, B., Elmfeldt, D., Julius, S., Ménard, J., Rahn, K. H., Wedel, H., and Westerling, S. (1998) Effects of intensive blood-pressure lowering and low-dose aspirin in patients with hypertension: principal results of the Hypertension Optimal Treatment (HOT) randomised trial. HOT Study Group. *Lancet,* **351,** 1755–1762.

[42] Weir, M. R., and Dworkin, L. D. (1998) Antihypertensive drugs, dietary salt, and renal protection: how low should you go and with which therapy? *Am. J. Kidney Dis.,* **32,** 1–22.

[43] Mogensen, C. E. (1999) Microalbuminuria, blood pressure and diabetic renal disease: Origin and development of ideas. *Diabetologia,* **42,** 263–285.

[44] Weber, M. A. (1991) Hypertension with concomitant conditions: The changing role of beta-adrenoceptor blockade. *Am. Heart J.,* **121,** 716–723.

[45] Loutzenhiser, R., and Epstein, M. (1990) Renal microvascular actions of calcium antagonists. *J. Am. Soc. Nephrol.,* **1,** S3–S12.

[46] Bakris, G., and White, D. (1997) Effects of an ACE inhibitor combined with a calcium channel blocker on progression of diabetic nephropathy. *J. Hum. Hypertens.,* **11,** 35–38.

[47] Demarie, B. K., and Bakris, G. L. (1990) Effects of different calcium antagonists on proteinuria associated with diabetes mellitus. *Ann. Intern. Med.,* **113,** 987–988.

[48] Hoelscher, D., and Bakris, G. (1994) Antihypertensive therapy and progression of diabetic renal disease. *J. Cardiovasc. Pharmacol.,* **23**(Suppl 1), S34–S38.

